# Multiple-Site Lichen Planus: An Italian Case Series of 44 Patients

**DOI:** 10.3390/jcm14217873

**Published:** 2025-11-06

**Authors:** Federico Bardazzi, Lidia Sacchelli, Giacomo Clarizio, Federica Filippi, Camilla Loi, Michelangelo La Placa

**Affiliations:** 1 Dermatology Unit, IRCCS Azienda Ospedaliero-Universitaria di Bologna, 40138 Bologna, Italy; federico.bardazzi@aosp.bo.it (F.B.); michelangelo.laplaca@unibo.it (M.L.P.); 2 Department of Medical and Surgical Sciences, Alma Mater Studiorum University of Bologna, 40126 Bologna, Italy

**Keywords:** erosive lichen planus, lichen planus, lichen sclerosus, oral lichen planus, vulvar lichen planus

## Abstract

**Background**: Lichen planus (LP) is a chronic immune-mediated inflammatory disorder affecting skin, mucosae, nails, and appendages, often with significant impact on quality of life. While associations between oral LP (OLP) and other localizations have been described, comprehensive analyses of patients presenting with multiple LP localizations remain limited. The aim of the study was describing the association of multisite LP among our patients in order to contribute to knowledge about this rare, but possible, clinical situation and its clinical implications in terms of follow-up. **Methods**: We conducted a retrospective observational study including 44 adult patients with histologically confirmed OLP and at least two additional LP subtypes. Data were collected at the joint dermatology–oral pathology clinic of Policlinico Sant’Orsola Malpighi, Bologna, between January 2022 and December 2024. Demographic characteristics, clinical manifestations, comorbidities, and therapeutic approaches were analyzed. **Results**: The cohort comprised 31 women and 13 men (mean age at first LP diagnosis: 56 years). All patients presented OLP, predominantly erosive (73%). During a follow-up, 39 patients developed three LP subtypes, and 5 patients developed four LP subtypes. Cutaneous LP was universal, while mucosal involvement included genital LP (*n* = 23), esophageal/pharyngeal/laryngeal LP (*n* = 8), and vulvar lichen sclerosus (*n* = 6). Nail LP was diagnosed in seven cases and frontal fibrosing alopecia in ten cases. Autoimmune comorbidities were frequent, including thyroiditis, psoriasis, systemic sclerosis, lupus, and Sjögren’s syndrome. First-line therapy consisted of topical and systemic corticosteroids, with adjuvant retinoids, cyclosporine, or immunosuppressants in refractory cases. No malignant transformation or dysplasia was detected during the observation period, and the mean follow-up period was 24 months (range: 12–36 months). **Conclusions**: Multisite LP is a complex, underrecognized condition requiring multidisciplinary management. OLP frequently represents the initial manifestation, followed by progressive involvement of cutaneous and mucosal sites. Regular full-body, oral, and genital examinations, together with tailored systemic treatments, are essential to prevent scarring sequelae and improve quality of life. Our findings highlight the need for heightened clinical vigilance and integrated care pathways for patients with multi-site LP.

## 1. Introduction

### 1.1. Definition and Epidemiology

Lichen ruber planus, also known as lichen planus (LP), is a chronic inflammatory, immune-mediated condition that can affect the skin, appendages, and different mucous membranes [1,2,3]. The term “lichen” comes from ancient Greek, meaning “tree moss”, due to its clinical appearance on skin, “ruber” due to the color of the lesions, mainly red-to-purple, and “planus” as the papules that characterize this disease are typically flat [1,4].

Worldwide prevalence of LP varies from 0.22 to 5%, according to different studies [3,4]. Among sexes, there is a slight female predilection (the male to female ratio is 1:2) [5]. The age of onset is between 30 and 60 years [3,6]. However, pediatric cases are described and are more common among Afro-descendant Americans [6].

LP represents the most common skin disease with oral involvement [5,7]. In fact, up to 77% of patients with cutaneous LP have an oral disease and about 15% of patients with oral lichen planus (OLP) present cutaneous LP [8].

### 1.2. Cutaneous LP (Table 1)

**Figure 1:**
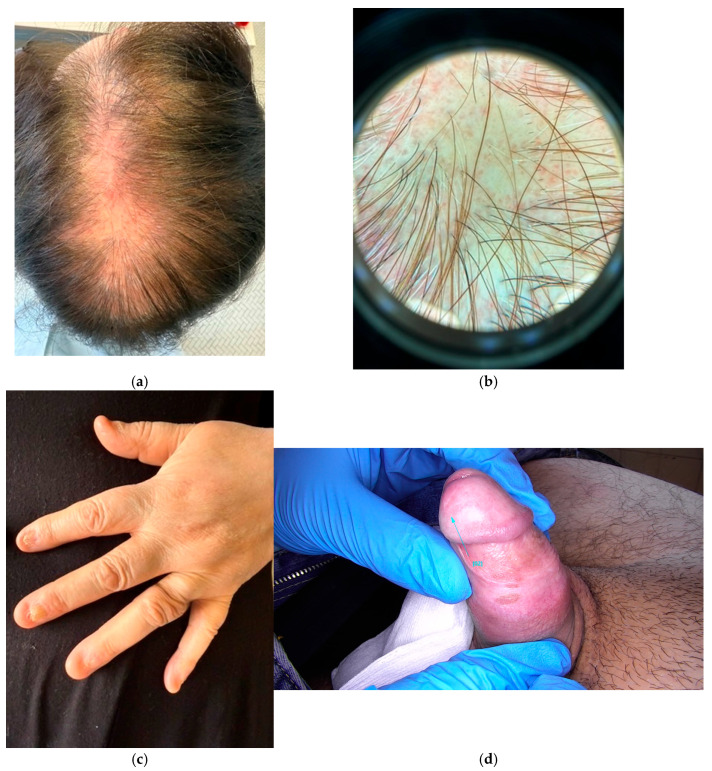
Lichen planopilaris (clinical and dermoscopy) (**a**,**b**). Alopecia of the scalp (**a**), dermoscopy showing fibrotic white dots, minimal erythema, and scarce hyperkeratosis perifollicularis (**b**) (20×, FotoFinder.Universe. versione 3.5.9.0). Nail lichen planus with complete alteration of the nail plate and dorsal pterygium (**c**). Genital lichen planus with annular shape on the glans (**d**).

Cutaneous LP encompasses various manifestations [4]: papular (classic form), hypertrophic [9], bullous [10], actinic [11], atrophic [12], linear [13], follicular (including the frontal fibrosing alopecia), lichen planopilaris (Figure 1a,b), Graham Little–Piccardi–Lassueur syndrome [4,14], pigmentosus [15] and, when involving the main folds, pigmentosus-inversus [16].

The cutaneous LP in its classic, papular form presents shiny, red–purple, flat-topped, and pruriginous papules mostly localized at the volar surface of the forearms, wrists, ankles, and other flexor areas of the body [4,17]. Wickham striae could be considered a diagnostic clue [18]. They appear as whitish structures in reticular patterns, corresponding to hypergranulosis at the histopathology [18]. They could be observed in the skin and oral mucosa but also on the scalp and nail folds [18].

The vesiculobullous subtype is characterized by blisters and vesicles developing above plaques: this form is in differential diagnosis with LP pemphigoids, a rare disease where LP and pemphigoids coexist [10]. On the male genital area (Figure 1c), namely on the glans and penile shaft, lichen presents in an annular pattern [4]. In darker skin types and in sun-exposed areas, a form-defined LP pigmentosus could develop [15]. Then, the atrophic subtype could be challenging if it is the only presentation of LP. However, more commonly, it represents the evolution of other subtypes of LP or the consequence of long-term corticosteroid therapy [12]. Follicular LP is one of the causes of cicatricial alopecia and could appear in alopecic patches or involving the frontline and eyebrows [4,14].

Other cutaneous LP forms are represented by palmoplantar LP mainly involving ankles, internal plantar arch [19], and nail LP [20]. This latter form ranges from 1 to 10% in adults and it has been described as having a higher frequency in children (up to 19%). Fingernails are more commonly involved than toenails and, likewise the follicular form, this LP could damage or even permanently destroy the nails if not properly treated [20]. Dorsal pterygium is typical of the classic form of nail LP and could be associated with fissuring of the nail plate, brittleness, and spontaneous onycholysis (Figure 1d) [21].

**Table 1:** Cutaneous LP subtypes.

Type	Clinical Characteristics	Typical Localization	Special Sites
Papular	Shiny, red–purple, flat-topped, and pruriginous papules	Volar surface of the forearms, wrists, ankles, and other flexor areas	On glans and penile shaft, annular pattern
Hypertrophic	Hyperkeratosic surface with possible scaling. Possible hyperpigmented or atrophic sequelae.	Lower limbs	
Bullous	Blisters and vesicles developing above plaques		
Actinic	Photo-induced form, sometimes with specific seasonality Hyperpigmented macules or papules with possible annular-arciform distribution	Photo-exposed areas	
Atrophic	Most commonly it represents the evolution of other longstanding forms		
Linear	Linear distribution of the classic papules along the Blaschko lines (Zosteriform distribution)	Limbs, especially lower; trunk; head and neck	
Follicular	Lichen plano-pilaris, Frontal Fibrosing Alopecia, Graham Little–Piccardi–Lasseur syndrome Forms of cicatricial alopecia involving the scalp and/or eyebrows		
Pigmentosus	Darker flat-topped and pruriginous papules	Sun-exposed area	When involving the intertriginous area, Inverse
Palmoplantar	Classical papules or hypertrophic lesions or erosive lesions	Ankles and internal plantar arch	
Nail	Dorsal pterygium: fissuring of nail plate, brittleness and spontaneous onycholysis	Fingernails are more commonly involved	

### 1.3. Mucous LP

Oral LP is the main mucosal district that could be involved, and it includes several subtypes: reticular, erosive, atrophic, papular, plaque-like, and bullous [4,22]. In 80–90% of cases, OLP is localized in the buccal mucosa, followed by the tongue and gums [23,24]. Clinically, reticular OLP is usually asymptomatic, while erosive and atrophic subtypes are associated with various degrees of life impairment, ranging from a burning sensation to severe pain and difficulty in feeding [25].

**Figure 2:**
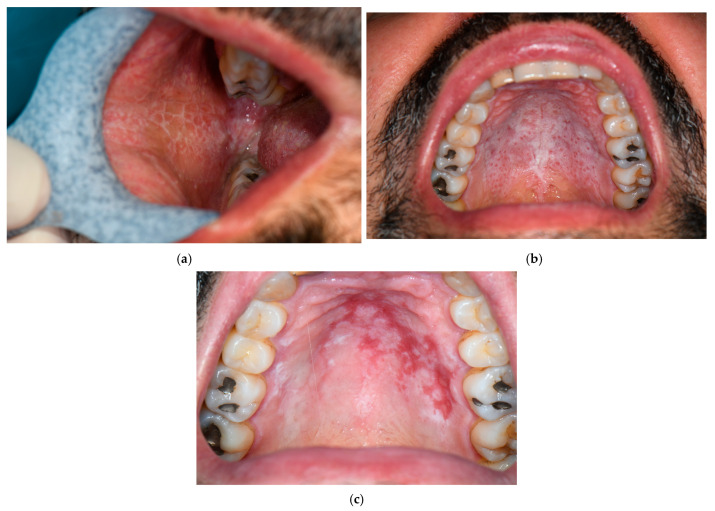
Reticular oral lichen planus involving the malar mucosa (**a**) and the hard palate mucosa (**b**). Erosive oral lichen planus (**c**).

The reticular OLP is the mucous counterpart of the classic cutaneous LP and, more often, it is overlooked (Figure 2a,b) [22]. By contrast, the erosive (Figure 2c) and the atrophic subtypes are associated with intense discomfort and pain [25]. When erosions occur, it is possible to see pseudomembranes covering the ulcers [4,25] and differential diagnosis from leukoplakia is mandatory [25]. The atrophic subtype primarily affects the attached gingiva [4].

Moreover, current evidence states that OLP is an oral potentially malignant disorder [26] that could evolve to squamous cell carcinoma (SCC) [3,26,27]. This risk is variable, ranging from 0.4% to 5% over periods of observation from less than 1 to 20 years according to the authors [17,28,29]. Malignant transformation seems higher in the longstanding atrophic and ulcerative forms of OLP [22], but also female sex and tongue localization seem to be predisposing factors [29].

A rare form of mucous LP is the vulvovaginal LP that could be erosive, papulosquamous, and hypertrophic [30]. Likewise, the OLP oral Lichen planus of this form could be associated with discomfort and pain, and there is a risk of potential scarring sequelae in the case of the erosive form [4,30]. Even more rare are the esophageal, ocular, and laryngeal LP [4,31].

### 1.4. Pathogenesis

Lichen planus is a T-cell-mediated autoimmune disease [1,4] characterized by a predominance of the T-helper 1 response with an activation of T cytotoxic lymphocytes, Natural Killer cells, and dendritic cells against basal keratinocytes inducing cell apoptosis both in skin and mucosa [20,32]. Moreover, pathogenetic pathways like the Fas ligand or granzyme B/perforin are involved [32]. These two proteins seems more abundant in OLP than in cutaneous LP [32]. LP pathogenesis involves cytotoxic T-cell-mediated basal keratinocytes apoptosis, supported by cytokine profiling in recent studies [33].

### 1.5. Diagnosis

Diagnosis of LP is usually based on clinical aspects and could be confirmed by histopathology in difficult cases [4].

At the histopathology (Table 2), LP presents a lichenoid interface dermatitis with a lymphohistiocytic infiltration of the dermo–epidermal junction and the upper dermis. This infiltrate is band-like with irregular epidermal hyperplasia, compact hyper- or ortho-keratosis and hypergranulosis [4,34].

Dermal papillae between elongated rete ridges are dome shaped; necrotic keratinocytes are observed at the basal layer, where the “Civatte bodies”, remnants of apoptotic keratinocytes, can also be found. Moreover, the vacuolar degeneration of the basal layer can induce subepidermal clefts (the so-called “Max Joseph spaces”).

In the atrophic LP, rete ridges are lost and dermal fibrosis is prominent.

Augmented melanophages in the papillary dermis induce residual post-inflammatory hyperpigmentation.

In the follicular LP, the lymphocytic infiltrate is localized in the peribulge area, in which there are follicular stem cells.

Then, in mucosal LP, histopathology is less specific, as the granular layer is absent and the rete ridges do not exhibit pronounced alterations.

Direct immunofluorescence could be useful for differential diagnosis [4,35]. For example, LP presents globular IgM deposition at the dermal–epidermal junction [35] while autoimmune bullous diseases classically show linear IgG and C3 deposition along the basement level [4]. Specifically, vesiculobullous LP and LP pemphigoides or vulvar LP and vulval lichen sclerosus are the most difficult to differentiate [4].

**Table 2:** Histopathological features of classical LP and specific differences in other forms (atrophic, follicular, and mucosal LP).

Classic LP	Atrophic LP	Follicular LP	Mucosal LP
Lichenoid interface dermatitis with a lymphohistiocytic infiltration of the dermo–epidermal junction and the upper dermis. Band-like infiltrate with irregular epidermal hyperplasia, compact hyper or orthokeratosis and hypergranulosis. Dome shaped dermal papillae in between elongated rete ridges; necrotic keratinocytes at the basal layer (“Civatte bodies”, remnants of apoptotic keratinocytes). Vacuolar degeneration of the basal layer can induce subepidermal clefts (“Max Joseph spaces”)	Loss of rete ridges and prominent dermal fibrosis	Lymphocytic infiltrate localized in the peribulge area	Absent granular layer and not pronounced alterations of the rete ridges

### 1.6. Associations of Multiple Forms

As above mentioned, cutaneous LP and OLP could be associated in a bidirectional manner [7,8].

Almost 25% of men with cutaneous LP showed genital LP [8].

Moreover, Belfiore et al. studied a population of 42 women with OLP, and they recorded a prevalence of 57% of vulval LP [36]. Before them, Pelisse et al. encoded the vulvovaginal-gingival syndrome where vulval, vaginal, and gingival mucosa were affected by LP [37]. Then, Cribier et al. described the male counterpart of this syndrome, the peno-gingival syndrome [38].

In recent years, the authors described a case series of 274 patients with cutaneous, genital, and oral LP, and of those patients, 37 presented more than one localization [6].

Thus, we decided to describe our case series of 44 patients presenting OLP and at least two other different types of LP. The aim of the study was to describe the association of multisite LP among our patients in order to contribute to knowledge about this rare, but possible, clinical situation and its clinical implications in terms of follow-up.

## 2. Materials and Methods

This study is a retrospective observational analysis based on real-world data collected from patients attending a tertiary referral center for inflammatory skin diseases. Patients were visited in the outpatients’ service of skin and oral diseases, where dermatologists cooperate with the stomatologist, specializing in inflammatory oral pathologies. The study population consisted of adult patients diagnosed with OLP, who were evaluated at the Dermatology Unit of the Policlinico Sant’Orsola Malpighi in Bologna between 1 January 2022 and 31 December 2024. The primary aim was to evaluate the subtype of ORL and the presence of other localizations of LP, both cutaneous and mucosa.

### 2.1. Inclusion Criteria

Participants were included in the study if they met all the following criteria:

- Age ≥ 18 years
- Confirmed clinical diagnosis of OLP by a board-certified dermatologist and dentist specialized in inflammatory oral pathologies
- Confirmed histopathological diagnosis of OLP
- Confirmed diagnosis of other forms of LP, cutaneous and/or mucosal
- Provided written informed consent for the retrospective use of anonymized clinical data. Data were anonymized and analyzed using descriptive statistics
- Available documentation reporting the clinical history of each patient

Patients lost at follow-up or refusing oral biopsy were excluded.

To note, diagnosis was clinical for cutaneous LP, except difficult cases, while OLP required both clinical and histopathological diagnosis.

The mean follow-up duration was 24 months (range: 12–36 months). Patients with missing follow-up data were classified as lost to follow-up. Data were included in the analysis only when a minimum follow-up of 12 months was available.

### 2.2. Ethical Considerations

The study received ethical approval from the Ethical Committee of the Policlinico Sant’Orsola Malpighi (protocol code “OBLI01, number 34-2023-OSS-AUSLBO, 18 January 2023”). The research was conducted in accordance with the principles of the Declaration of Helsinki (1964) and subsequent revisions. All participants provided written informed consent for the anonymous use of their clinical and histopathological data for research purposes.

The study encompasses data collection from 31 October 2017 to 31 December 2024.

Patients have given informed written consent to clinical images, as in our clinical practice.

## 3. Results

A total of 44 adult patients of both sexes with OLP were included in the study. The majority of the cohort was represented by female patients, 31 out of 44 (70%). Mean age at the diagnosis of the first LP was 55.5 years (±SD 16), males were slightly younger than females (53.8 vs. 55.8 years).

Mean disease duration was 10.5 years (±SD 5.76); while mean follow-up time was 24 months (ranging from 12 to 26 months).

### 3.1. Comorbidities Autoimmune and Non-Autoimmune (Table 3)

In regard to autoimmune comorbidities, in our cohort we found:

- 5 cases of Hashimoto’s thyroiditis
- 2 cases of undifferentiated connective tissue disease
- 3 cases of plaque psoriasis
- 1 case of Sjögren syndrome
- 1 case of autoimmune atrophic gastritis
- 1 case of morphea
- 1 case of systemic sclerosis
- 1 case of systemic lupus erythematosus

**Table 3:** Autoimmune comorbidities among patients included in the study. Number of patients and percentage are shown.

Autoimmune Comorbidity	Number	Percentage
Hashimoto’s thyroiditis	5	11.3
Undifferentiated connective tissue disease	2	4.5
Plaque psoriasis	3	6.8
Sjögren Syndrome	1	2.7
Autoimmune atrophic gastritis	1	2.7
Morphea	1	2.7
Systemic sclerosis	1	2.7
Systemic lupus sclerosus	1	2.7

### 3.2. Multiple-Site Lichen Planus

All 44 patients included in the study presented OLP, and at least one of two other types of lichen planus, either cutaneous and/or mucosal (Table 4).

Typically, during each visit we examined the skin, oral cavity, and genital area.

Moreover, patients were asked about symptoms like dysphagia and dry cough, especially if it recently appeared. If these symptoms were present, patients were prescribed a specialistic visit with a gastroenterologist or otolaryngologist.

In regard to OLP, 32 patients out of the 44 presented an erosive form as first localization, while 12 developed other forms of OLP over time, namely 9 reticular and 3 plaque-like OLP.

During the follow-up, all patients presented cutaneous involvement as lichen ruber planus.

Of the total of 44 patients:

- 8 developed a rare form of mucosal LP (4 esophageal, 3 pharyngeal, 1 laryngeal)
- 23 presented ano-genital LP, 6 as lichen sclerosus (5 females and 1 male), 10 as LP with annular pattern (11 males), and 6 as erosive vulval LP
- 10 had an involvement of the scalp with frontal fibrosing alopecia
- 7 presented nail LP
- 1 developed a severe erosive form of palmoplantar LP with scarring sequelae

None of the patients presented during the follow-up the evolution to leukoplakia or dysplasia, and, in any case of doubt of transformation, an oral biopsy was performed. The mean follow-up duration was 24 months (range: 12–36 months).

Of the total, 39 patients presented 3 different types of LP, all including OLP, during the observation period while 5 patients presented 4 different types of LP.

**Table 4:** Demographic characteristics of patients enrolled in the study: the numbers indicate the chronology of each diagnosis of lichen. Age indicates the age at the diagnosis of the first form of lichen. (Pt, patient; OLP, oral lichen planus; LRP, lichen ruber planus; ELP, esophageal lichen planus; PLP, pharyngeal lichen planus; LLP, laryngeal lichen planus; FFA, frontal fibrosing alopecia; GLP, genital lichen planus; LS, lichen sclerosus; NLP, nail lichen planus; PPLP, palmoplantar lichen planus).

Pt	Sex	Age	OLP	LRP	ELP	PLP	LPL	FFA	GLP	LS	NLP	PPLP
1	M	58	1	2					3			
2	F	69	1	2	3			4				
3	M	37	1	2					3			
4	M	63	1	2					3			
5	M	68	1	2					3			
6	M	42	1	2					3			
7	M	66	2	1					3			
8	F	56	2	3				4		1		
9	M	26	1	2							3	
10	M	55	2	3					1			
11	F	27	1	2						3		
12	F	40	2	1					3			
13	F	56	2	3				1			4	
14	F	42	2	1						3		
15	F	73	1	3				2			4	
16	F	64	1	2				3				
17	F	73	1	2				3				
18	F	78	1	2					3			
19	F	50	1	3				2				
20	F	49	1	3							2	
21	M	47	1	2					3			
22	M	72	1	3					2			
23	F	67	2	3				1				
24	F	67	2	1	3							
25	F	30	2	1							3	
26	F	62	1	3				2				
27	F	57	2	3							1	
28	M	79	1	2					3			
29	M	49	1	3					2			
30	F	70	3	2	1							
31	F	78	1	3				2				
32	F	69	1	2					3			
33	F	69	1	2					3			
34	F	40	2	1			3					
35	F	75	2	1		3						
36	F	42	1	2					3			
37	F	30	1	2					3			
38	F	44	1	2							3	
39	F	57	1	2		3						
40	F	52	1	2	3							
41	F	48	3	2						1		
42	M	47	2	3						1		
43	F	70	2	3						1		4
44	F	60	1	2		3						

### 3.3. Therapeutic Approaches to Multi-Site LP (Table 5)

Topical and/or systemic corticosteroids were used as the first line treatment in all 44 patients, according to the Italian guidelines [21].

In 12 cases out of 44, systemic anti-fungal drugs were used in the prevention of oral or genital candidiasis.

In 13 cases out of 44, retinoids were prescribed both in topical and systemic administration. Namely, topical tretinoin and/or systemic acitretin.

In refractory cases or in those that were cortico-dependent, other treatments were introduced as follows:

- 13 cases of cyclosporine A (CyA) both topical and/or systemic
- 3 cases of mycophenolate mofetil
- 1 case of methotrexate
- 4 cases of dapsone
- 1 case of systemic nicotinamide
- 1 case of adalimumab and apremilast

Treatment response was evaluated according to lesion regression and symptom improvement on follow-up. Data shown in the table describe clinical response at the last follow-up during the study.

**Table 5:** Therapeutic approaches and clinical responses at the last follow-up for each patient involved in the study. (Pt, patient; CSt, topical corticosteroids; CSs, systemic corticosteroids; TIMs, topical immunomodulators; CyA t, topical Cyclosporine A; CyA s, systemic Cyclosporine A; MMF, Mycophenolate mofetil; MTX, Methotrexate).

Pt	CSt	CSs	TIMs	Anti-Fungal	Tretinoin	Acitretin	CyA t	CyA s	MMF	MTX	Dapsone	Nicotinamide	Apremilast	Adalimumab	Improved	Stable	Refractory
1	x	x	x	x											x		
2	x	x	x	x				x								x	
3	x	x		x											x		
4	x	x		x											x		
5	x	x				x									x		
6	x														x		
7	x														x		
8	x		x	x												x	
9	x	x														x	
10	x	x		x											x		
11	x	x														x	
12	x	x			x			x								x	
13	x	x	x													x	
14	x	x							x		x						x
15	x	x	x									x				x	
16	x	x	x	x												x	
17	x	x	x						x		x						x
18	x	x														x	
19	x	x	x												x		
20	x	x														x	
21	x	x													x		
22	x	x									x				x		
23	x	x	x		x	x		x								x	
24	x	x			x	x		x								x	
25	x	x														x	
26	x	x	x													x	
27	x			x						x							x
28	x	x														x	
29	x	x			x		x									x	
30	x	x		x												x	
31	x	x	x	x	x			x								x	
32	x				x										x		
33	x			x				x							x		
34	x	x			x			x								x	
35	x	x				x		x	x							x	
36	x	x		x											x		
37	x	x			x		x								x		
38	x	x														x	
39	x	x		x				x			x						x
40	x	x				x										x	
41	x	x													x		
42	x	x													x		
43	x				x	x		x					x	x			x
44	x	x			x	x		x								x	

## 4. Discussion

### 4.1. Muti-Sites LP

The association of multiple-site forms of LP is now well-known, but first descriptions date back only 40 years [6,8,36,39].

The first description of the association of genital and oral LP was published by Pelisse et al. in 1982 as vulvovaginal-gingival syndrome (VVG) [37]. This syndrome presents erosive vulvovaginitis and gingivitis [37,39]. Then, a few years later, Cribier et al. described the male counterpart [38]. Typically, VVG affects middle-aged female patients, as in our cohort where mean age was 56 years. The authors emphasized the importance of clinical examination of both the oral and genital areas during consultations to avoid overlooking LP involvements that could be developed over time [37,39]. This is in line with our data where 12 patients out of 44 presented OLP during the follow-up period.

More recently, Belfiore et al. showed a prevalence of 57% of vulval LP in patients with OLP [36]. To note, of these patients, 92% did not report relevant symptoms before examination [36]. Thus, the authors concluded that vulval involvement may be underestimated, underlining the importance of genital examination in patients with OLP [36].

Not only vulval LP may be associated with OLP but also lichen sclerosus (LS), as reported by Janovska et al. [40]. In their case series of 86 patients, 50% received diagnosis before the diagnosis of OLP, as in our cohort where OLP was the first diagnosis for 25 patients out of 44.

In contrast with these results, Corazza et al., in their prospective study involving 300 women with vulval LS, stated a very low incidence of oral association [41]. In particular, none of the patients presented oral LS and 6 women out of 300 had a histologically confirmed diagnosis of OLP [41]. The apparent opposition to our results with these may be due to the selection of the patients’ cohort.

Many authors agree that OLP may be the first clinical presentation of LP, and other sites may be involved subsequently [36,37,40]. Hence, careful follow-up of oral, cutaneous, and mucosal sites is critical to detect new localizations and prevent neoplastic transformation. Malignant transformation should not be overlooked in both situations. OLP has an estimated transformation of 1.4% over time [40], especially in its erosive form [23].

Additionally, vulval LS is associated with this risk, especially in untreated cases, with a lifetime risk of 3 to 5% [40]. Furthermore, as emphasized, some cases of vulval LP or LS may be asymptomatic, and our examination is mandatory along with cooperation between other physicians (stomatologists and gynecologists) [36].

To note, even though the malignant evolution of the OLP is an option, notably in erosive OLP, we did not report any cases of transformation. This could be due to the short follow-up observed in this study as authors report rates of malignant evolution over the course of up to 20 years [28,30].

Prompt treatment of LP is mandatory to ameliorate the quality of life of patients (In case of OLP, above all the erosive one), and to reduce the risk of atrophic or scarring sequelae, and further malignant transformation.

Thus we believe it is important to ask patients the right questions in order to rule out rare mucosal involvements, as seen in our cohort. Esophageal, pharyngeal, and laryngeal LP are rare [32]. In particular, esophageal LP has an estimated prevalence of 0.19% and it is more common in middle-aged women [32]. Authors report a delay in the diagnosis of years due to misinterpretation of symptoms like GERD (gastro-esophageal reflux disease) [32]. For this reason, it is important to consider a screening endoscopy in case of dysphagia or odynophagia that is imputable to OLP [32]. Moreover, this rare form is associated with a significant risk of SCC [32]. This evolution seems rapid (within one year after the diagnosis) and relatively frequent (6.1%) [42].

### 4.2. Association with Other Autoimmune Diseases

The most common autoimmune diseases associated with LP are thyroid disease, vitiligo, systemic lupus erythematosus, Sjögren’s syndrome, psoriasis, and type I diabetes mellitus [43,44].

Data from the literature are in line with our cohort.

Furthermore, the authors recommend screening for autoimmune diseases in case of vulval LS in order to rule out thyroid disease and pernicious anemia [45].

Thus, it might be advisable to screen our LP patients for autoimmune diseases.

With the current knowledge, the precise mechanism responsible for these associations is still unclear. Some authors found no strong association with many autoimmune diseases (like psoriasis or hypothyroidism) but reported a statistically significant association with atopic dermatitis, diabetes mellitus type I, and ankylosing spondylitis [44]. However, other authors focused on autoimmune thyroid disease and found various HLAs and potential external triggers commonly shared by LP, LS, and thyreopathy [46]. More data are needed for conclusive results.

### 4.3. Therapeutic Approaches (Table 6)

The therapeutic approach depends on the severity and the extent of the disease. In the majority of cases, topical corticosteroids were used, in line with the Italian guidelines [23].

In cases of multiple sites and severe disease, systemic corticosteroids were associated with topical ones. Alone or in combination with corticosteroids, retinoids were used, namely acitretin and tretinoin.

In refractory cases, CyA was administered both topically and systemically with favorable results in 8 patients out of 44.

In other cases, second- and third-line treatments were used such as methotrexate, mycophenolate mofetil, dapsone, and nicotinamide.

Then, anti-TNF-a (adalimumab) and apremilast were used in the case of the LP with atrophic evolution on the palmoplantar area as rescue therapy, with little success.

Additionally, a special mention should be made about promising emerging regenerative treatments such as injectable platelet-rich fibrin (i-PRF) and platelet-rich plasma (PRP) [47].

These treatments are interesting because of their ability to mitigate symptoms like pain and to promote tissue healing, especially in OLP [47].

**Table 6:** Therapeutic approaches in each patient enrolled in the study. (Pt, patient; CSt, Corticosteroids topical; CSs, Corticosteroids systemic; CyA t, Cyclosporine A topical; CyAs, Cyclosporine A systemic; MMF, Mycophenolate mofetil; MTX, Methotrexate; TIMs: Topical Immune Modulators).

Pt	CSt	CSs	TIMs	Anti-Fungal	Tretinoin	Acitretin	CyA t	CyAs	MMF	MTX	Dapsone	Nicotinamide	Apremilast	Adalimumab
1	x	x	x	x										
2	x	x	x	x				x						
3	x	x		x										
4	x	x		x										
5	x	x				x								
6	x													
7	x													
8	x		x	x										
9	x	x												
10	x	x		x										
11	x	x												
12	x	x			x			x						
13	x	x	x											
14	x	x							x		x			
15	x	x	x									x		
16	x	x	x	x										
17	x	x	x						x		x			
18	x	x												
19	x	x	x											
20	x	x												
21	x	x												
22	x	x									x			
23	x	x	x		x	x		x						
24	x	x			x	x		x						
25	x	x												
26	x	x	x											
27	x			x						x				
28	x	x												
29	x	x			x		x							
30	x	x		x										
31	x	x	x	x	x			x						
32	x				x									
33	x			x				x						
34	x	x			x			x						
35	x	x				x		x	x					
36	x	x		x										
37	x	x			x		x							
38	x	x												
39	x	x		x				x			x			
40	x	x				x								
41	x	x												
42	x	x												
43	x				x	x		x					x	x
44	x	x			x	x		x						

## 5. Conclusions

Lichen planus is a chronic immune-mediated disease that may involve skin, appendages, and mucosae [2].

For this reason, LP may have multiple forms of presentation with various grades of impairment of quality of life: an itch in the cutaneous LP or pain in the OLP or in the genital localization. Reducing or resolving symptoms, along with prevention of the cicatricial sequelae (especially among patients with mucosal LP, genital or esophageal) is mandatory to guarantee a favorable quality of life for these patients.

Moreover, it is important to not overlook the potential risk of the malignant evolution of some forms of OLP or genital LP.

Furthermore, LP is associated with a higher risk of other autoimmune diseases that should be ruled out [45,46,47].

Then, LP may have multiple sites of localization that clinicians should consider, investigate, and treat properly. They may develop through years of follow-up or may be present at the first visit. An integrated approach to these patients, according to our opinion, is very important and necessary for comprehensive care.

Authors suggestions for a holistic take in charge of patients with LP (at the first consultation and during follow-ups) in order to guarantee early recognition of multi-site LP

- Detailed anamnesis ruling out symptoms of dysphagia or odynophagia
- Total body examination, including oral cavity and genital area
- Blood tests: complete blood count, basic metabolic panel (to rule out diabetes, dyslipidemia, and impaired liver function), and blood urea nitrogen (to rule out impaired renal function)
- Autoimmune diseases: blood tests according to clinical suspicion along with TSH reflex, ANA reflex, and parietal cells antibodies
- Multidisciplinary approach to patients involving dentist, gynecologist, gastroenterologist, or otorhinolaryngologist
- Perform skin and mucosal biopsy if necessary to rule out leukoplakia
- Check-ups are recommended at least once a year

### Limitations of the Study

Limitation of the study could be represented by its retrospective design, the small sample of patients included, and the single center study.

## Data Availability

The raw data supporting the conclusions of this article will be made available by the authors on request.

## Abbreviations

The following abbreviations are used in this manuscript:

LP	Lichen Planus
OLP	Oral Lichen Planus
SCC	Squamous Cell Carcinoma
FFA	Frontal Fibrosing Alopecia
CyA	Cyclosporine A
VVG	Vulvovaginal-Gingival syndrome
LS	Lichen Sclerosus
GERD	Gastroesophageal Reflux Disease
